# Facile preparation of Cu–Fe oxide nanoplates for ammonia borane decomposition and tandem nitroarene hydrogenation†

**DOI:** 10.1039/d1ra04175d

**Published:** 2021-09-06

**Authors:** Guoqiang Wang, Chuanjun Wang, Hao Zhang, Youle Liu, Jing Xu

**Affiliations:** College of Chemistry and Material Science, Shandong Agricultural University Tai'an 271018 P. R. China wangchuanjun@sdau.edu.cn

## Abstract

A facile substrate involved strategy was used to prepare Cu–Fe LDO (layered double oxide) nanoplates. The material exhibited good-efficiency for decomposition of ammonia borane (AB) in alkaline methanol solution. Significantly, the material also demonstrated excellent catalytic performance in the reduction of various nitroarenes by coupling with AB hydrolysis in a one pot tandem reaction, and gave excellent yields of the corresponding amine products.

## Abstract 2

CuFe oxide nanoplates were developed which displayed good activity and stability toward AB solvolysis. The material transformed to Cu/Fe_2_O_3_ while catalyzing AB reduction and in a tandem system reduced nitro compounds to amines in substantial yield.

## Graphical abstract

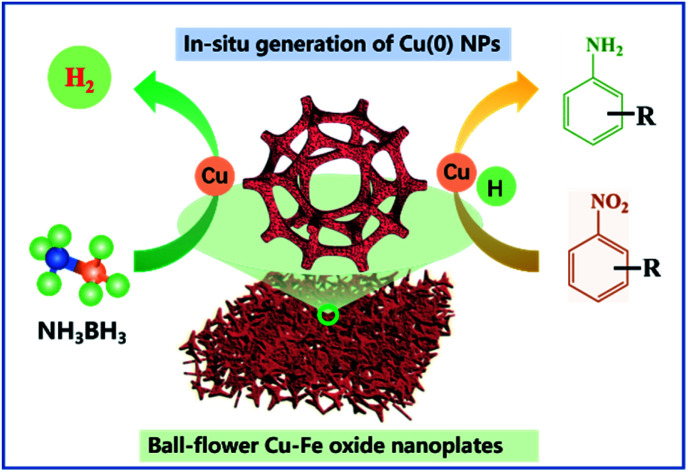

The increased human exploitation of fossil fuels has caused serious environmental problems and depletion of resources, which makes it necessary to develop new types of green and sustainable alternative energy sources.^1,2^ Hydrogen has attracted great attention and is likely to become the main source of energy in the future.^3,4^ As chemical hydrogen storage materials, B–N compounds generally have high hydrogen densities.^5^ Among which, ammonia borane (AB) has been widely studied due to its excellent hydrogen storage and production capacity; it is non-toxic, harmless and can be dissolved in solvents such as methanol and so on.^6,7^ Great efforts have been made to investigate the hydrolysis of AB using noble and transition metal based catalysts.^8^ Nevertheless, the continuous search for economical and easily obtainable catalysts has been never-ending.^9,10^

On the other hand, functionalized aromatic amines are important reaction intermediates and raw materials for production of value-added compounds, pharmaceuticals and agricultural chemicals.^11,12^ They are generally synthesized by hydrogenation of the nitroarenes under pressurized H_2_ with transition metal based catalysts. The exploitation of alternative hydrogen storage source with efficient and stable catalysts are still key to achieve high catalytic performance.^13^ Although some noble-metal based catalysts (such as Pt,^14^ Rh^15^ and Pd^16^) can achieve good selectivity by alloying with other metals or coupling with supporting materials, the high price and scarcity limit their applications. Therefore, the development of earth-abundant transition metals catalysts with high activity and chemical selectivity is still very necessary.^17^ Meanwhile, although AB as hydrogen reservoir is not strongly reducing enough to initiate the hydrogenation of nitroarenes, the utilization of transition metal based catalysts which expediently decompose AB (NH_3_·BH_3_ + 2H_2_O → NH_4_^+^ + BO_2_^−^ + 3H_2_↑)^18^ while catalysing hydrogenation of nitroarenes to target amines under *in situ* generated H_2_ in a one-pot tandem system provides a promising strategy to realize the reduction processes.^19,20^

To our knowledge, few work have explored the application of transition metal based LDHs (and LDHs derivatives) for AB decomposition and nitroarenes hydrogenation.^21^ The layered double hydroxides (LDHs) is a type of material composed of layers of di- and trivalent metal cations coordinated to hydroxide anions, the layered structure allows exposing a large number of active sites.^22,23^ It has demonstrated promising activity for oxygen evolution reaction (OER),^24^ nitrogen reduction reaction (NRR).^25^ Due to its adjustable multiple metal centres and large surface areas derived from its lamellar structure, LDHs makes ideal candidate to serve as either synergistic catalyst or as supporting catalyst precursor.^26^ In general, upon heat treatment LDHs would transform into LDOs (layered double oxides),^27^ which maintains the layered structure with exposed active sites that can serve as ideal candidate for transition metal oxides catalysed reactions. These intrinsic properties led us to conceive that transition metal based LDHs (and LDOs) could be potentially desirable cost-effective material for AB hydrolysis and nitroarenes reduction.

Herein, as a proof of study we report a fast and substrate involved preparation of Cu–Fe LDOs nanoplates as high-efficiency catalyst for tandem decomposition of AB and hydrogenation of various nitroarenes. We use iron foam (IF) as the supporting substrate, and add H_2_O_2_ to accelerate the redox process^28^ to quickly synthesis uniform CuFe oxide nanoplates tightly attached to the surface of iron foam at room temperature for the first time. The resultant CuFe LDOs can release H_2_ efficiently from alkaline (OH^−^) AB methanol solution. Significantly, the material also showed excellent catalytic performance in tandem reduction of various nitroarenes through coupling with AB hydrolysis. It gave very handsome yield of corresponding amine products within short reaction time. Characterization were performed on CuFe LDOs before and after AB hydrolysis and revealed that the synthesized CuO/Fe_2_O_3_ (LDOs) were *in situ* converted to Cu/Fe_2_O_3_ after reaction, active hydrogen *in situ* absorbed on Cu(0) nanoparticles renders the high selective activity for amine formation. This work demonstrates the feasibility of using well-structured transition metal LDHs as raw material to effectively achieve new reduction reactions.

The Cu–Fe LDOs was synthesised in a one pot reaction by using Fe foam as substrate introduced into a H_2_O_2_ solution containing Cu^2+^ (4 mmol) and Fe^3+^ (0.2 mmol) ions (ESI). Fe foam participated in and accelerated the reaction while H_2_O_2_ decomposed and precipitated the target material. A substantial amount of O_2_ bubbling and heat release was observed during reaction, it took only a few minutes to successfully grow the product that tightly binds on the surface of Fe substrate, with an observable colour change from silver white to brown. The reaction mechanism was proposed as follow: initially Fe^3+^ reacts with Fe foam to generate Fe^2+^ on surface; Fe^2+^, Fe^3+^ and H_2_O_2_ make up the classic Fenton reaction system in which Fe^2+^ is oxidized by H_2_O_2_ to Fe^3+^; then Fe^3+^ and Cu^2+^ co-precipitates on iron foam to form Fe(OH)_3_ and Cu(OH)_2_ LDHs as OH^−^ increases;^29^ LDHs was eventually converted to LDOs under the internal heat releasing condition. Control experiments revealed that in the absence of either Cu^2+^ or Fe^3+^, reaction proceeded very slowly.

The material was subsequently characterized by scanning electron microscopy (SEM), X-ray diffraction (XRD), and Raman spectroscopy, transmission electron microscopy (TEM), X-ray photoelectron spectroscopy (XPS) as well as Fourier transform infrared spectroscopy (FTIR). As shown in Fig. 1b, the iron foam after reaction was covered by a uniformly distributed layer of rugged materials. Magnified SEM images of the surface materials (Fig. 1c and d) revealed the formation of chrysanthemum shaped ball-flowers with diameters around 2 μm. The ball-flower is composed of bundles of numerous thin and long nano-leaves, and therefore confer the material with large surface area and abundant exposed active sites (Fig. 1d). Elemental mapping images of different ball-flowers clearly shows the presence of Fe and Cu elements in the sample, which are in an approximate concentration ratio 4 : 1 based on EDX analysis (Fig. 1e and S2 in ESI†). XRD characterization was performed with the as prepared Cu–Fe sample self-grown on the iron foam for many times, however, distinct diffraction peaks were not obtained. A weak peak which can be ascribed to Cu(111) was observed in some tests, which was due to metal replacement reaction between Fe foam and added Cu^2+^ (Fig. 2a). Therefore, the material could be amorphous or that a too small thickness of the material was synthesized. To provide more evidence, we further performed TEM experiments (Fig. 3a), the result showed the formation of ball-flower material corresponding to the SEM images in Fig. 1, and the transparent thin-plates proved the small thickness of layers. In addition, TEM-mapping were also performed, the result demonstrated the uniform distribution of Fe, Cu and O elements on the material (Fig. 3b–f). HRTEM clearly showed the stacking of very thin layered plates usually typical of layered structure, and the various complex lattice fringes revealed the material as poly-crystalline (ESI, Fig. S3†).

**Fig. 1:**
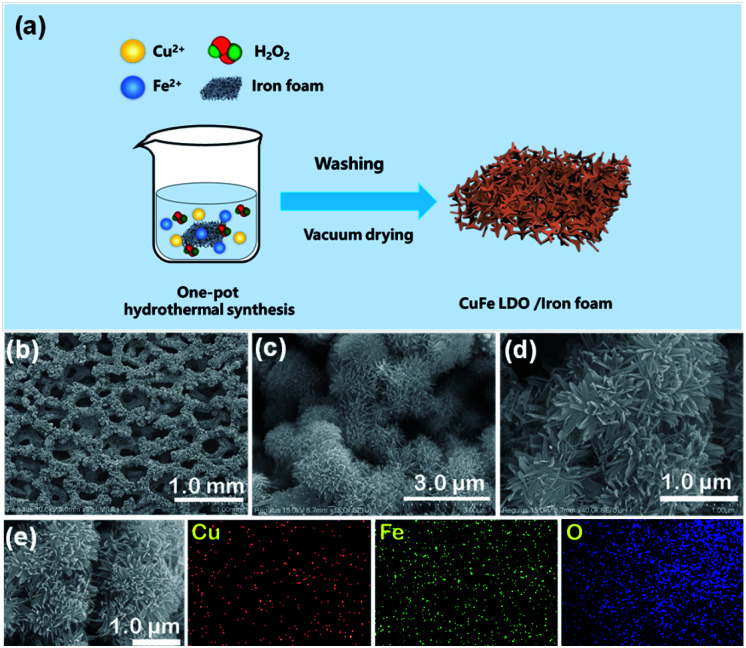
(a) Scheme for CuFe LDO synthesis. (b) SEM image of CuFe LDOs generated on iron foam; (c and d) magnified SEM images showing the formation of flower-clump like layered structure. (e) CuFe LDOs nanoplates bundle and corresponding elemental mapping of Cu, Fe and O.

**Fig. 2:**
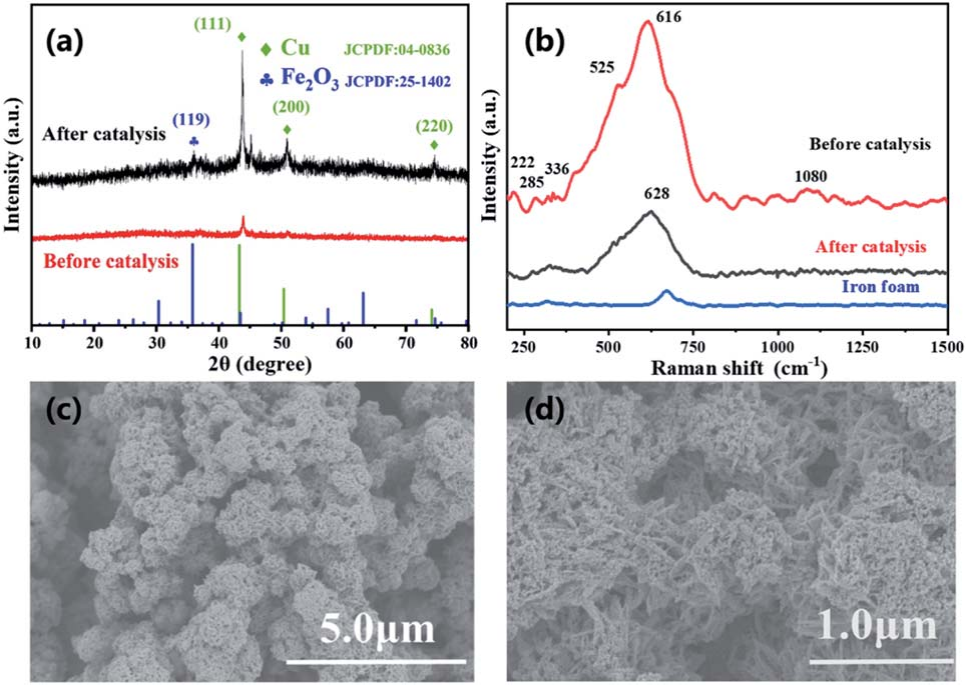
(a) XRD of CuFe LDOs after reaction; (b) Raman spectra of CuFe LDOs before and after catalysis; (c and d) magnified SEM images showing the production of nanoparticles on the flower-clump like nanoplate structure.

**Fig. 3:**
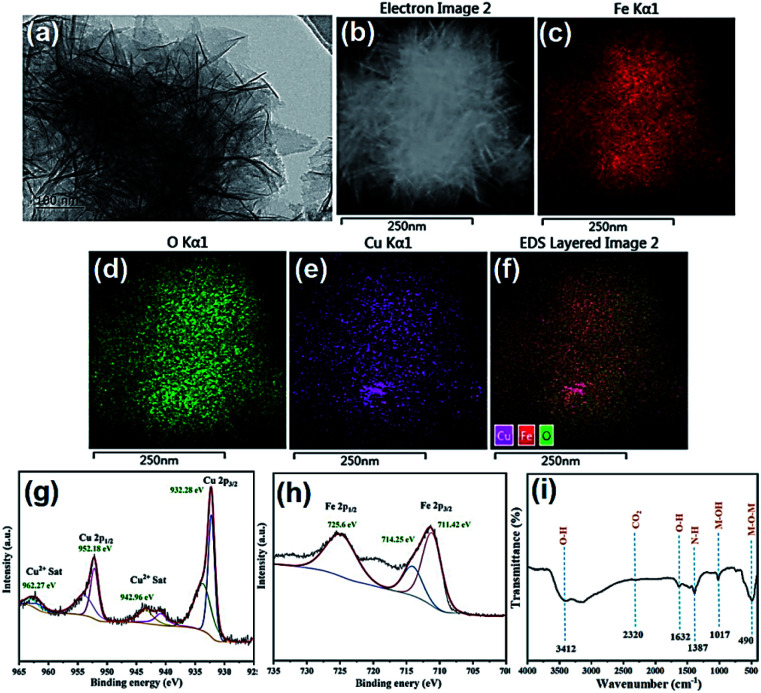
(a) TEM; (b–f) TEM-mapping images; (g and h) Cu 2p and Fe 2p XPS spectra; (i) FTIR spectra of Cu–Fe LDOs.

Furthermore, Raman spectrum provided evidence for the formation of material (Fig. 2b), analysis of the material formed on Fe foam displayed the main peak of CuO at 616 cm^−1^, along with three minor peaks at 222, 285, 336 cm^−1^. The weak peaks appeared at 1080 and 525 cm^−1^ are indication of residue Cu(OH)_2_ according to literature, which further proves the transformation process of LDH to LDO as described in above material preparation part.^30^ The Raman spectra showed mainly the formation of CuO oxide, the peaks of iron species were not obvious as also happened to Raman of iron foam (Fig. 2b). Therefore, XPS experiments were also performed to provide more information. The XPS spectra where peaks corresponding to C 1s, Cu 2p and Fe 2p were detected (Fig. 3g–h). For Cu 2p, two typical peaks were located at 952.18 and 932.28 eV, corresponding to Cu 2p_1/2_ and Cu 2p_3/2_. The other two peaks at 942.96 and 962.27 eV were the satellite peaks of Cu 2p. These results indicates that the copper in the material appeared as Cu^2+^ with the outermost electron configuration of 3d^9^.^31^ For Fe 2p, the XPS peak of Fe 2p_1/2_ was located at 725.60 eV, and the peak of Fe 2p_3/2_ was two splitting peaks at 711.42 and 714.25 eV, indicating that iron appeared as Fe^3+^ with the outermost electron configuration of 3d^5^. In addition, the position of the binding energy at 714.25 eV indicated the presence of Fe–O on the surface of the composite.^31^

FTIR spectra were also recorded using KBr method (Fig. 3i), which coincides with that of Cu–Fe LDH as reported in literature. The peaks at 1017 and 490 cm^−1^ were ascribed to Cu–O–Fe stretching vibration and metallic bond vibration (M–O), respectively. The results indicated that the as-prepared material possessed a hydrotalcite-like structure, and the copper and iron atoms in the metal layer were connected by an oxygen atom.^32^

From the above characterization results, we may conclude as to the formation of layered double oxide materials of Cu–Fe oxides.

Subsequently, the material was used as a model catalyst and tested for AB decomposition for H_2_ production at room temperature. The produced gas was identified using gas chromatography and collected volumetrically with self-built setup. A 0.5 mol L^−1^ NaOH methanol solution was used based on control experiments (ESI, Fig. S5†) and report that OH^−^ could activate the B–N bond.^33^ The molar ratio of Cu(NO_3_)_2_ and Fe(NO_3_)_3_ precursors used in material preparation were varied to find an optimized proportion that gave best H_2_ release activity.

As can be seen from Fig. 4a, when Cu^2+^concentration was varied from 0 to 6 mmol with fixed Fe^3+^ (0.2 mmol), the system showed increased activity for H_2_ generation from AB (0.06 g in 25 mL H_2_O) decomposition. It should be noted the material formed very slowly with only Fe^3+^ precursor did not show any activity, suggesting the essential role of Cu for LDO generation as well as dehydrogenation of AB. However, it was found that a thick layer of material was formed at high precursor concentration (Cu^2+^/Fe^3+^: 6/0.2) which easily fell off from iron foam and were difficult to handle for subsequent tests. Therefore, the catalytic system (Cu^2+^/Fe^3+^: 4/0.2) which generates catalyst with a tighter binding was chosen as model for most tests as well as characterizations. The kinetics of AB dehydrogenation by the model CuFe LDO was further studied under varying substrate concentrations.

**Fig. 4:**
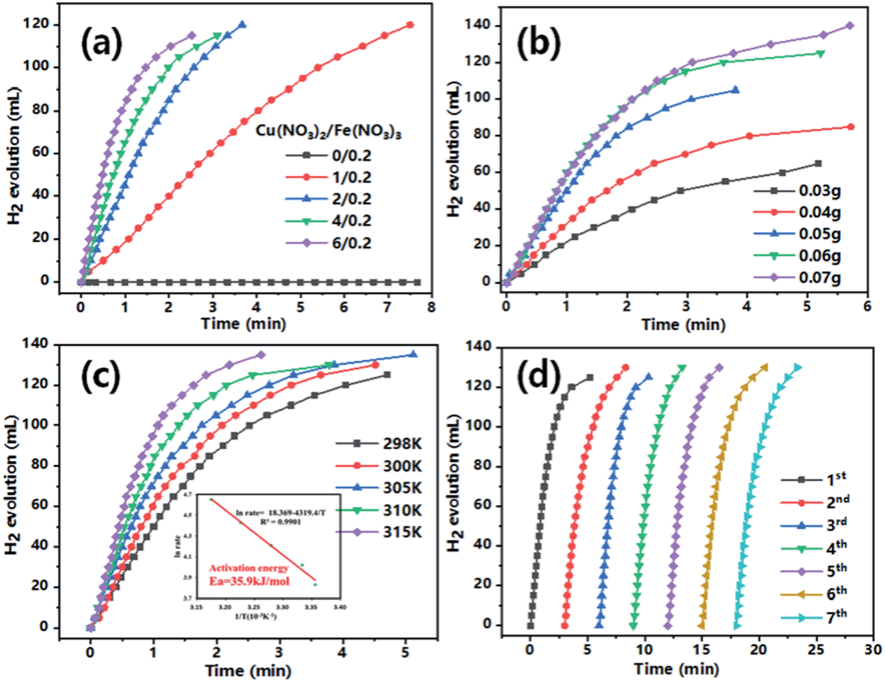
H_2_ evolution curves of AB solvolysis catalysed by catalyst (a) formed at different substrate ratios at 300 K; (b) with different AB amount by model LDO (added Cu^2+^/Fe^3+^: 4/0.2); (c) as a function of temperature in solvolysis of AB (0.06 g) with CuFe LDO (added Cu^2+^/Fe^3+^: 4/0.2; inset illustrations is logarithmic plot of H_2_ generation rate *versus* 1/*T*). (d) Recycling test of CuFe LDO catalyst. (All reactions were conducted in 5 mL of 0.5 mol L^−1^ NaOH methanol solution.)

As demonstrated in Fig. 4b, the H_2_ generation rate increased with increasing AB amount in solution (0.03–0.07 g), a higher reaction rate resulted from intimate contacts between catalyst and substrate. The maximum reaction rate was observed with 0.06 g AB, as system with a higher amount of 0.07 g added AB did not exhibit an even higher reaction rate. The reactions were performed in a temperature range of 298–315 K (Fig. 4c) To get the activation energy (*E*_a_) of AB dehydrogenation by CuFe LDO. It is obvious that the reaction rate is enhanced upon increasing temperature. The Arrhenius plot of ln rate *vs.* 1/T is plotted (Fig. 4c, inset). The *E*_a_ is calculated to be approximately 35.9 kJ mol^−1^. The material after one reaction was subjected to XRD, Raman, SEM characterizations. XRD revealed the formation of Cu/Fe_2_O_3_ after reduction reaction, the peaks appeared at 43.7°, 50.9° and 74.6° can be attributed to the (111), (200) and (220) crystal planes of Cu (JCPDS no. 04-0836). A small peak observed at 36.2° was evidence of the (119) crystal plane of Fe_2_O_3_ (JCPDS no. 25-1402).^20,21,34^

Furthermore, Raman characterization revealed the appearance of a peak at 628 cm^−1^, which was right shifted compared to CuO (616 cm^−1^) and was ascribed to the reduced Cu(0) in reference to literature (Fig. 2b).^21,30,35^ In addition, the morphology of LDO after reaction was characterized by SEM, it was discovered that after AB solvolysis there were spherical nanoparticles *in situ* formed and attached on the surface of nanosheets clumps. The change in nanostructure coordinates with the reduction transformation of Cu species, and the generated nanoparticles was related to the Cu catalyst. The BET analysis indicates the lowering in porosity due to formation of nanoparticle clumps (Fig. S6†). Therefore, it was evident from these data that the CuFe LDO material acted as precursor in AB solvolysis reaction, and reduction of CuO by ammonia borane would send forth the Cu(0) nanoparticles *in situ* from the nanosheets.

The generated Cu/Fe_2_O_3_ after one reaction was collected and gently washed with deionized water, it was then subjected to recycling tests for AB decomposition. As can be seen from Fig. 4d, the reclaimed catalyst showed good recyclability and reusability. There is no apparent decrease in AB catalytic activity even after 7 repeated tests. The SEM image also provide evidence that the material maintained morphology after the recycling tests (Fig. S7†). Very importantly, when Cu/Fe_2_O_3_ generated from LDO after the initial AB solvolysis was used for the second tests, the Cu/Fe_2_O_3_ showed faster reaction rate than the LDO in the initial test (Fig. S8†). This result is due to the transformation of CuO/Fe_2_O_3_ to Cu/Fe_2_O_3_ catalyst in initial test, which in the second test Cu readily exists to catalyse AB decomposition.

Additionally, these interesting results led us to exploit the use of CuFe LDO as catalyst for the hydrogenation of common functional aryl nitro compounds using AB as hydrogen source in a tandem reaction system, by simply adding nitro compounds to the AB solution. The activity and selectivity of this catalyst is comparable to noble metal nanocatalysts, which has meaningful significance for application to the industrial scale preparation of aromatic amines.

As can be seen from Table 1, nitrobenzene was chosen as the model substrate for optimizing reaction conditions. In the system for AB decomposition under room temperature, the reaction of AB (0.97 mmol), the model CuFe LDOs on iron foam, and nitrobenzene (0.1 mmol) in 0.5 M NaOH methanol (5 mL) 96% yield within only 20 min of reaction time (entry 1^*a*^). Control experiments indicated that both LDOs and AB are indispensable for achieving nitro reduction in high yield. Importantly, replacing methanol with deionized water led to a decreasing yield for aniline production upon 20 min of reaction. Several cycles of catalytic nitrobenzene reduction experiments were carried out using the same sample, and the decrease in yield is mainly attributed to the falling off of the catalyst from iron foam during the cleaning process (Fig. S10d†).

**Table 1:** CuFe LDO-catalyzed nitroarene hydrogenation

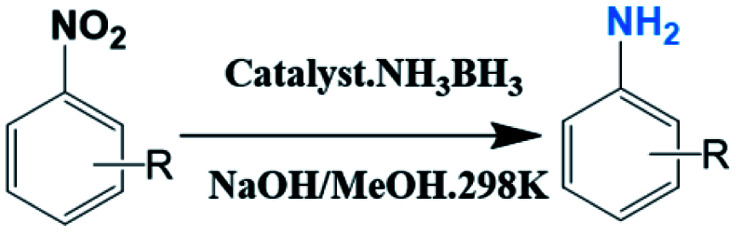					
Entry	Substrate	Product	*t* (min)	Yield (%)	Selectivity (%)
1^a^	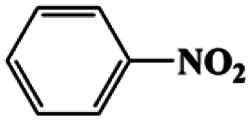	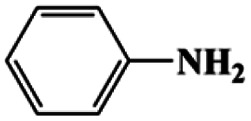	20	96	>99
1^b^			60	0	0
1^c^			60	8	>99
1^d^			20	91	>99
2^a^	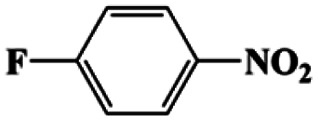	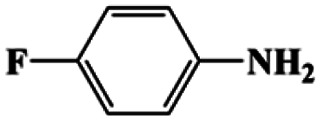	20	96	>99
3^a^	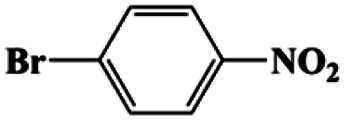	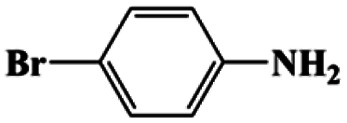	20	95	>99
4^a^	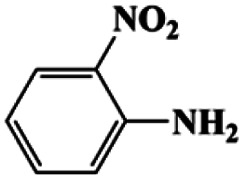	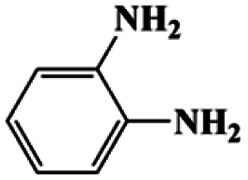	20	92	>99
5^a^	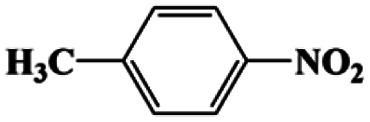	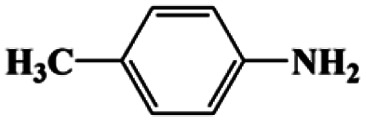	20	85	>99
6^a^	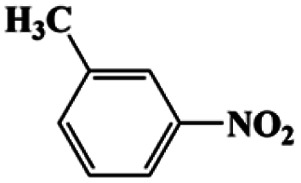	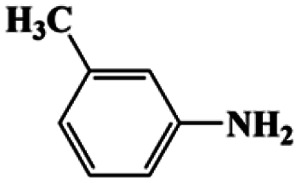	20	86	>99

a Catalyst CuFe LDOs. Reaction condition of *a*: 0.1 mmol of nitroarene, 0.97 mmol of NH_3_BH_3_, 5 mL of 0.5 M NaOH methanol solution, 298 K.

b Without adding NH_3_BH_3_.

c Without adding any catalyst.

d Use deionized water as solvent.

Moreover, the common substrate scope of this reaction was probed to reveal the activity of CuFe LDOs toward various nitro-compounds with electron-donating groups, including Me and NH_2_, as well as electron-withdrawing groups, such as halogens. Generally, very high yields of corresponding –NH_2_ compounds were obtained upon 20 min of reaction based on NMR results (Fig. S9†) as well as UV-vis spectroscopic measurements (Fig. S10†). Compounds with halo substituents, including F and Br were well tolerated in and its substituted nitroarene could be converted into its corresponding aniline in high yield and selectivity. Notably, the aromatic compound bearing both –NO_2_ and –NH_2_ groups could be reduced to diamine. Since neither H_2_ gas nor Cu catalyst alone possess activity for nitroarenes reduction, it is therefore likely that the catalyst facilitates the effective formation of active hydrogen species (H˙ or hydride) from AB, which absorbs on generated Cu surface to effectively mediate the reduction reaction.^20^ Future work were expected to provide more information.

## Conclusions

In this work, we developed a facile method to prepare CuO/Fe_2_O_3_ LDOs with layered nanoplate structure. The material displayed good activity toward AB solvolysis. The material *in situ* transformed to Cu/Fe_2_O_3_ while catalysing AB reduction, and Cu is identified as the active catalyst. Additionally, under optimized conditions, various substituted amines were obtained from the corresponding nitro compounds in a tandem system upon reduction of AB. This work demonstrates the practical feasibility of utilizing transition metal based LDHs (and LDOs) as potentially desirable cost-effective supporting material as well as catalyst precursor for achieving reduction reactions.

## Conflicts of interest

There are no conflicts to declare.

## Author contributions

C. J. Wang and J. Xu conceptualized the study and led the project. G. Q. Wang performed the bulk of catalyst preparation, characterization, and catalytic tests. H. Zhang and Y. L. Liu contributed to catalytic tests. All authors contributed to the writing of the manuscript and data analysis.

## Supplementary Material

RA-011-D1RA04175D-s001
