# Supplementary material for: Facile preparation of Cu–Fe oxide nanoplates for ammonia borane decomposition and tandem nitroarene hydrogenation
Source: RSC Adv. 2021 Sep 6;11(48):29920–4. doi: 10.1039/d1ra04175d (PMC9040926; doi:10.1039/d1ra04175d)
Supplement: RA-011-D1RA04175D-s001 [file RA-011-D1RA04175D-s001.pdf]

Electronic Supplementary Material (ESI) for RSC Advances.
This journal is © The Royal Society of Chemistry 2021

Electronic Supporting Information

Facile preparation of Cu-Fe oxide nanoplates for Ammonia Borane decomposition and tandem nitroarenes hydrogenation

Guoqiang Wang,[a] Chuanjun Wang,[a*] Hao Zhang,[a] Youle Liu,[a] Jing Xu [a*]

[a] College of Chemistry and Material Science, Shandong Agricultural University, Tai'an 271018, P. R. China

Experimental Methods

Materials:

All chemicals and solvents were of analytical grade and used directly.

Fe foam (1 ×1 cm) with a thickness of 1.6 mm was purchased from Kunshan Jiayisheng electronics Co., Ltd. Copper(II) nitrate hydrate($Cu(NO_3)_2$ $3H_2O$, 99%), Iron(III) nitrate nonahydrate ($Fe(NO_3)_2$ $9H_2O$, 98.50%), Borane-ammonia complex (NH_3BH_3, 97%) were obtained from Maknlin Chemistry Co., Ltd. Potassium Bisulfate(KHSO4), nitrobenzene, p-fluoronitrobenzene, p-bromonitrobenzene, p-nitrotoluene, 2-nitroaniline, m-nitrotoluene were purchased from Aladdin Chemistry Co., Ltd. N-(1-naphthyl) ethylenediamine dihydrochloride, Zinc powder, absolute ethanol, methanol, H_2O_2 (30 wt%), NaOH, HCl were obtained from Sinopharm Chemical Reagent Co., Ltd. Sodium sulfamate was purchased from Tianjin Guangfu Fine Chemical Research Institute. Sodium nitrite ($NaNO_2$) was purchased from Wuxi Yatai United Chemical Co., Ltd.

Characterization

All samples characterized by X-ray diffraction patterns (XRD) (conventional Bragg-Brentano geometry) were using a Rigaku Smartlab SE X-ray diffractometer with Cu K αradiation (λ= 1.5406 ˚A). Raman spectroscopy was performed on a Horiba Scientific XploRA™PLUS Laser Raman Spectrometers. IR was performed using a Bruker Tensor II IR Spectrophotometer. The morphology of the samples was characterized by Hitachi Regulus8100 field emission scanning electron microscope (SEM). The conversion rate of nitroaromatic compounds were determined by measuring the absorbance at 545nm with UV spectrophotometer (Shimadzu 2700). ^{1}H NMR analyses were performed on a BRUKER AVANCEIIIHD500 NMR spectrometer. BET surface area was determined by the Specific surface area and porosity analyzer (micromeritics, Gemini VII 2390). X-ray photoelectron spectroscopy (XPS) was performed on an ESCALAB 250Xi system and a monochromatic Al K αX-ray source with a power of 250 W was used. All XPS spectra were calibrated by referring the carbon 1 s peak to 284.8 eV. Transmission electron microscopy (TEM) images were measured on a JEOL JEM-2100F machine with accelerated voltage of 200 KV.

Synthesis of CuFe NP/IF

Pieces of iron foam (1 ×1 cm) were cleaned by 1 M HCl solution, ethanol and deionized water with the assistance of ultrasonication for 20 min. Firstly, 10 mL H_2O_2 and 8 mL deionized water were added to a 250 mL flask. Then 0.9664 g $Cu(NO_3)_2$ (4 mmol) was dissolved in this flask, which formed a blue-green solution. At last, 2 mL $Fe(NO_3)_3$ (0.1 M) was added to this flask and the pretreated iron foam (silvery) was transferred into it, simultaneously. At room temperature (25 °C), 200 s later, the color of the iron foam surface changed from silvery white to brown. Then the sample was taken out, washed several times by deionized water and dried in vacuum drying oven.

Catalytic AB hydrogen release activity tests

The bottle of a two-necked round-bottom flask (25 mL) was sealed one end with a rubber cap and connect the other end to a gas measuring cylinder filled with water through a gas scrubber. In the process of testing, stirring with a magnetic stirrer, constant-temperature bath was employed to maintain the reaction temperature and a thermometer was employed to detect the temperature of the reaction system. Dissolve 0.1 g NaOH in 4 mL of methanol solution in a reaction flask containing a catalyst and a magnet, and then assemble the reaction flask and the measuring device. Dissolve 0.06g AB in 1mL methanol solution in another vial. When the temperature reaches 300K and stabilize, use a syringe to inject the AB methanol solution into the reaction flask through the rubber stopper. At this time, the reaction

system is 0.5mol/L NaOH methanol solution the reaction was supposed to start immediately. The volume of H_2 was measured by drainage method. For the durability test, after the catalytic dehydrogenation of AB ceased, the catalyst was collected, washed with deionized water, ethanol. The recovered catalyst was then used in the next run of solvolysis reaction at 298 K.

Tandem reaction dehydrogenation of AB and hydrogenation of nitrobenarenes over CuFe NP/IF

CuFe NP/IF, 0.1g NaOH, 4 mL of methanol and 0.1 mmol of nitroarene were placed in a two-necked round-bottomed flask (25 mL) under magnetic stirring. Then, purge with nitrogen for 5 minutes under slow agitation to remove air from the reactor and dissolved oxygen in the solution. The water bath was heated to 298K under magnetic agitation. The reaction got started when 1 mL of AB solution (30mg) was added into the flask.

Determination of aniline compounds

The amount of aniline compounds in the reaction solution was determined by reductive azo spectrophotometry. Aniline compounds under acidic conditions (pH1.5-2.0) and nitrite diazotization, and then coupled with N-(1-naphthalyl) ethylenediamine hydrochloride, to produce purple dye, spectrophotometric determination, the measurement wavelength is 545nm. As a control, add zinc powder to the reacted compound in an acidic solution containing copper sulfate to generate nascent hydrogen, which can completely reduce the incompletely reacted nitrobenzene compounds to aniline.

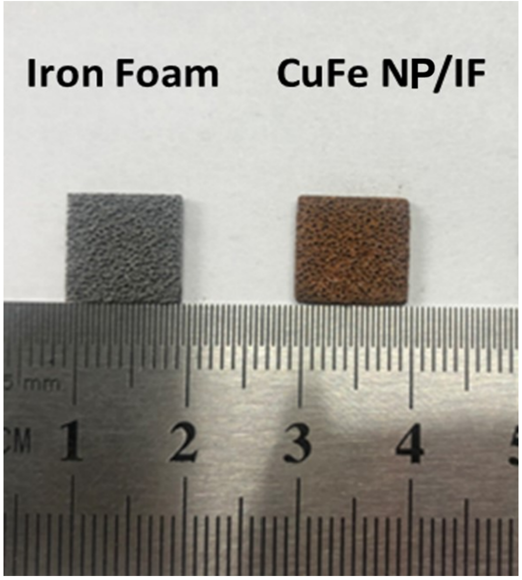

Figure S1. Photograph of the iron foam and CuFe NP/IF.

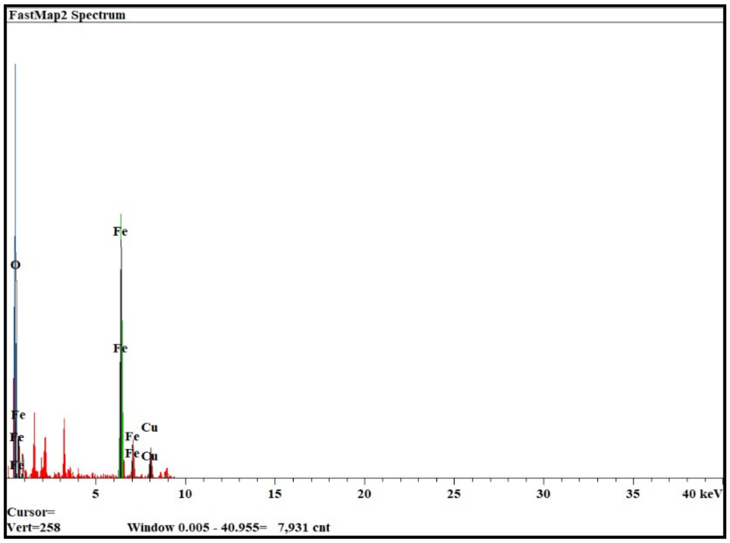

Elt.	Line	Intensity (c/s)	Conc.	Units	Error 2-sig	MDL 3-sig	
O	Ka	33.54	29.658	wt.%	2.739	3.364	
Fe	Ka	54.40	55.596	wt.%	2.514	1.111	
Cu	Ka	7.65	14.745	wt.%	2.005	1.620	
			100.000	wt.%			Total

kV	20.0
Takeoff Angle	35.0°
Elapsed Livetime	39.2

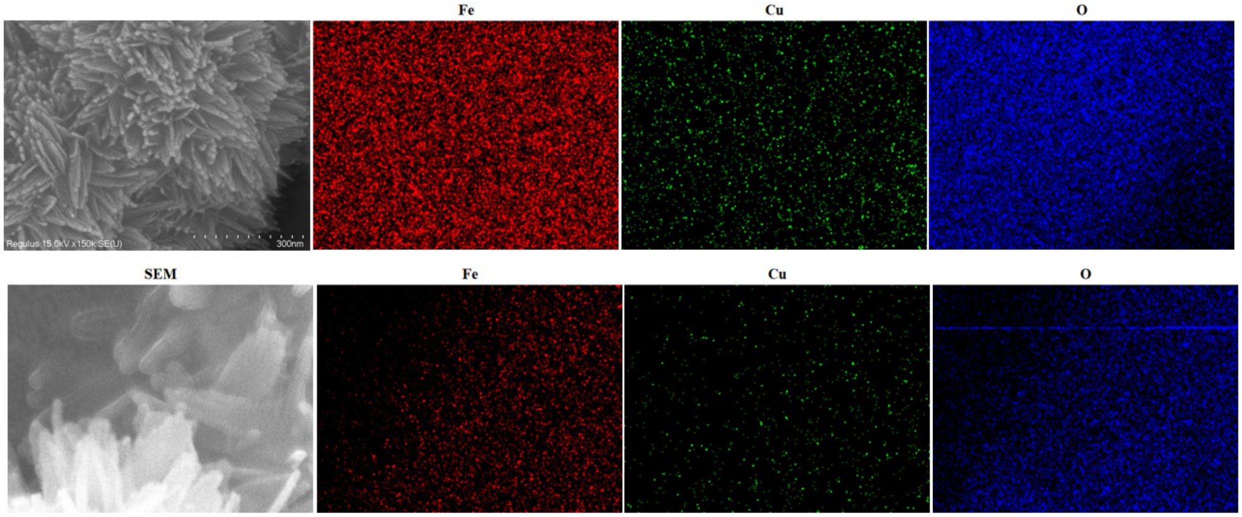

Figure S2. Corresponding EDX spectra of prepared CuFe NP/IF in Figure 1; and SEM-mapping at higher magnifications.

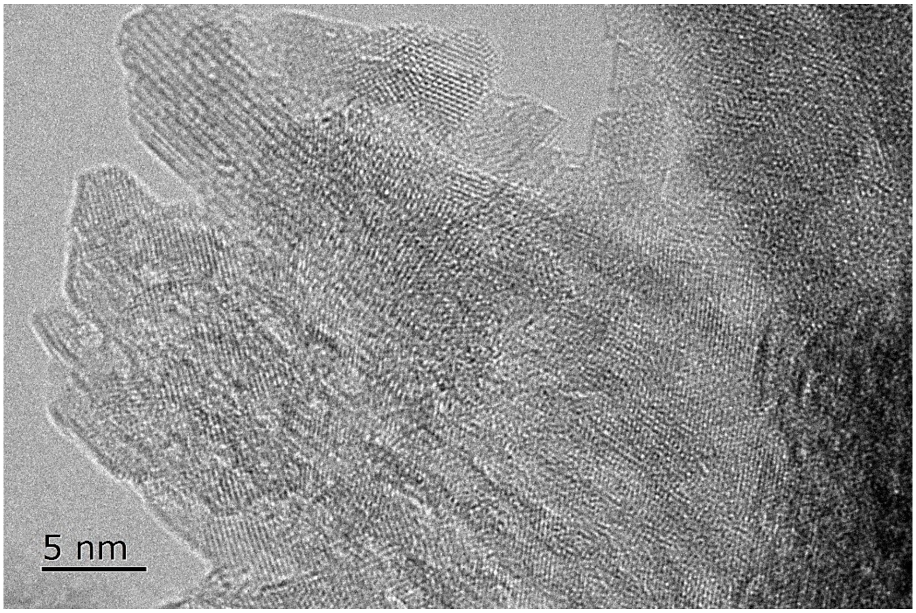

Figure S3. HRTEM spectra of prepared CuFe NP/IF。

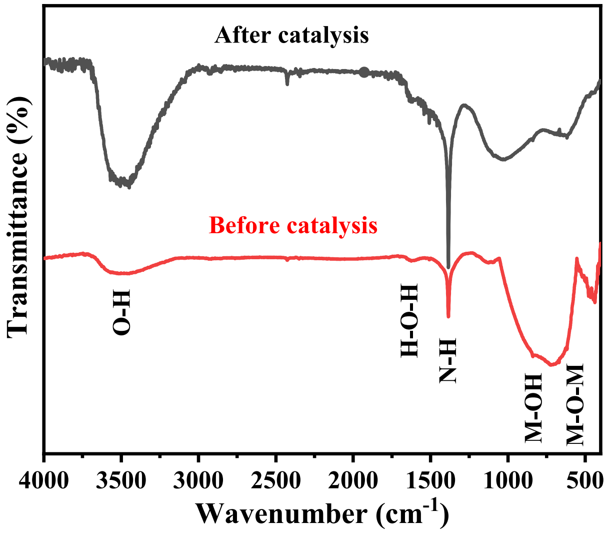

Figure S4. FT-IR spectra before and after CuFe NP/IF catalysis.

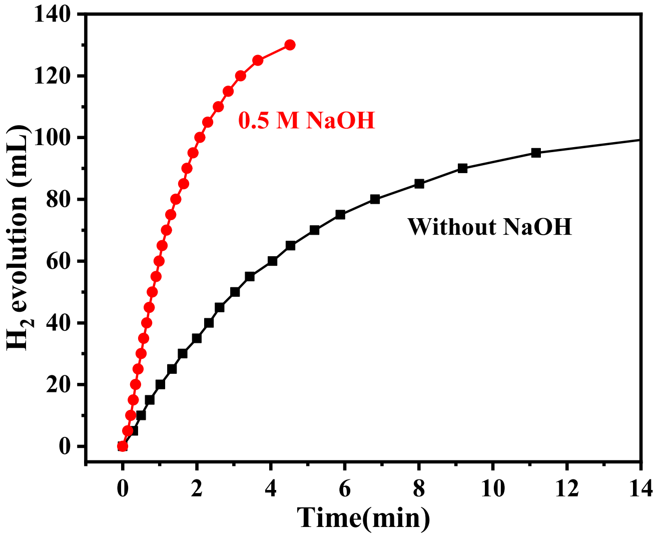

Figure S5.H_2 evolution curves of the solvolysis of AB catalyzed by CuFe NP/IF under different solution conditions at 298K.

Sample	BET surface area (m^2/g)
**Before**	21.8186
**After**	19.7523

Figure S6. BET of LDO before and after catalysis.

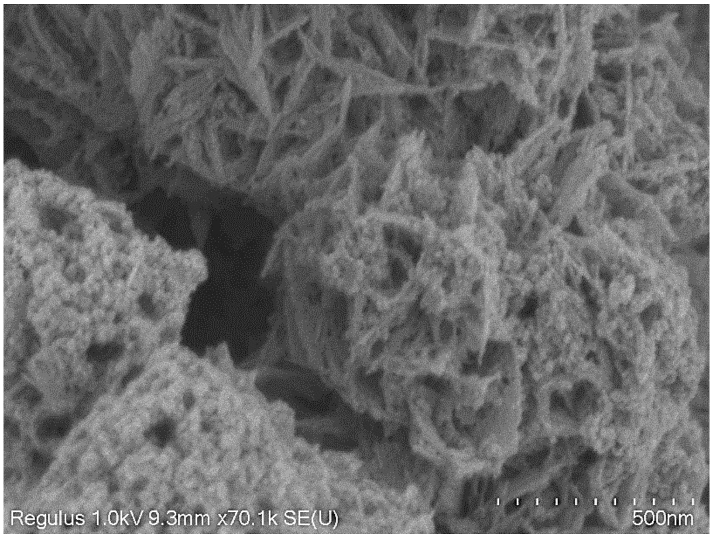

Figure S7. SEM of LDO after catalysis.

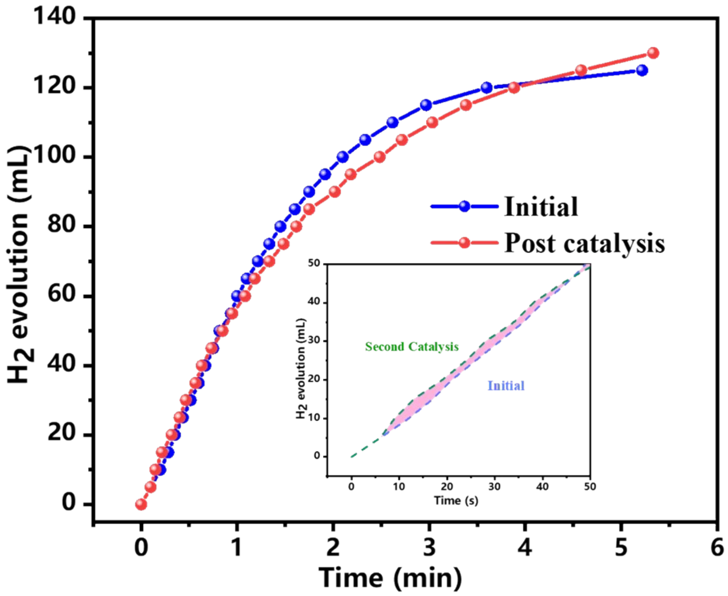

Figure S8. First and second catalysis of Cu-Fe LDO for AB.

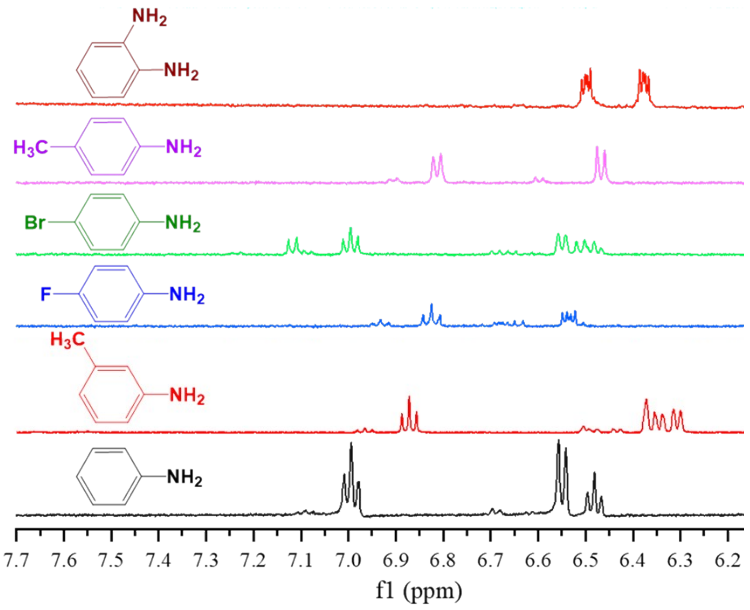

Figure S9. ^{1}H NMR spectra of the reaction solution with the samples in DMSO-d^6 as the solvent.

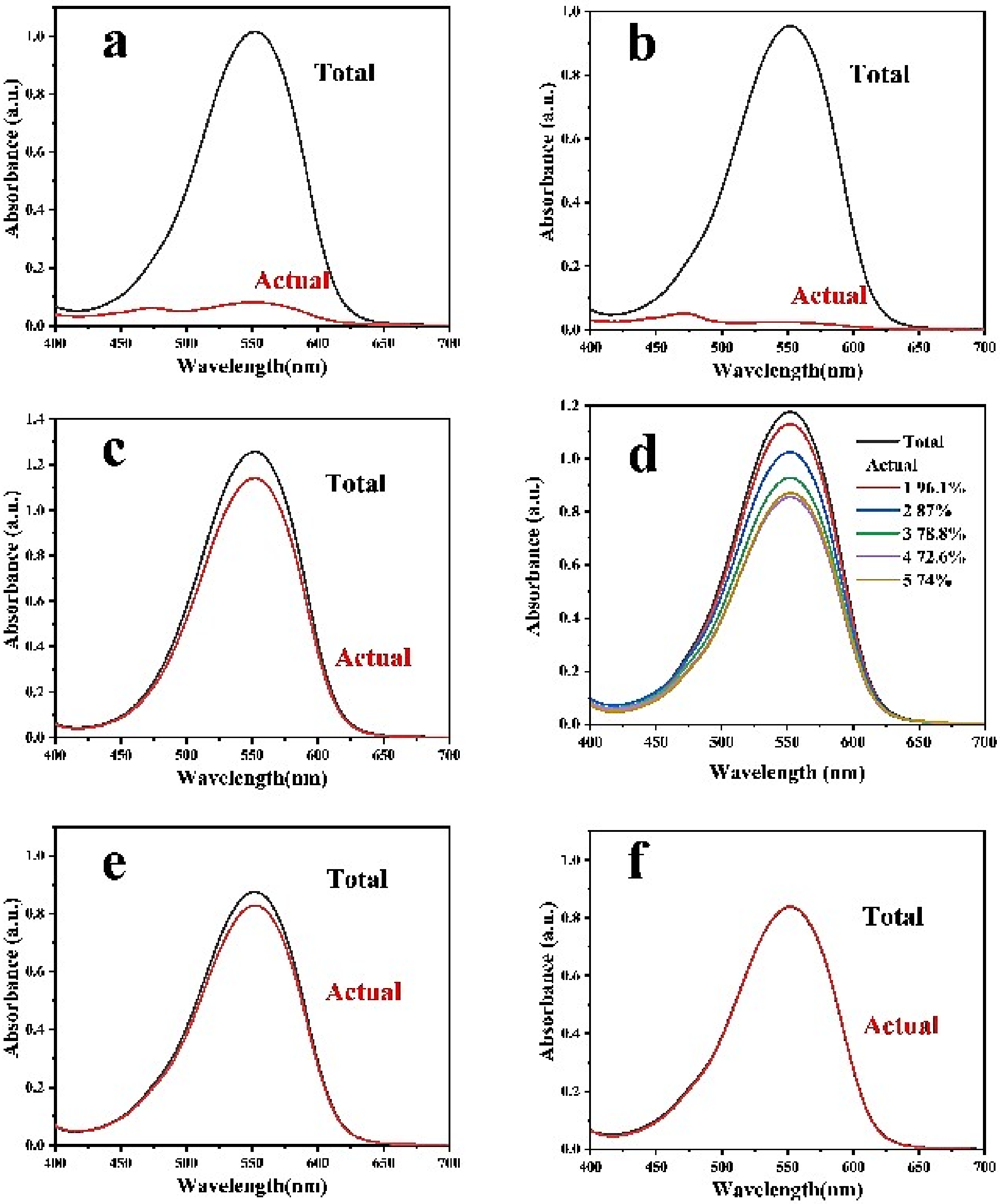

Figure S10. UV absorption spectrum of (a) without adding any catalyst; (b) without adding NH_3BH_3; (c) using deionized water as solvent; (d) recycling test of tandem; (e) 20 min reaction and (f) prolong reaction time.

(All reactions were conducted in 5 mL of 0.5 mol L^{-1} NaOH methanol solution, 298K, 0.1 mmol of nitrobenzene, 20min).
